# Positive affect as a developmental mediator of early adversity and internalizing psychopathology

**DOI:** 10.1111/jcpp.70104

**Published:** 2026-01-13

**Authors:** Jamie L. Hanson, Dorthea J. Adkins, Isabella Kahhale, Sriparna Sen

**Affiliations:** ^1^ Learning, Research & Development Center University of Pittsburgh Pittsburgh PA USA; ^2^ Department of Psychology University of Pittsburgh Pittsburgh PA USA

**Keywords:** adversity, affective disorders, emotion, risk factors, resilience

## Abstract

**Background:**

Early life adversities (ELAs) including experiences such as abuse, neglect, and household dysfunction are strongly linked to psychopathology; yet, the developmental pathways connecting ELA to externalizing and internalizing psychopathology remain unclear. While most research has focused on threat and negative affect, positive emotions may represent a critical but understudied mechanism linking ELA to mental health outcomes.

**Methods:**

Using data from the Adolescent Brain Cognitive Development (ABCD) study, we examined positive affect trajectories across six timepoints spanning childhood through adolescence (ages 9–10 to 12–13). We employed person‐centered trajectory‐based clustering to identify distinct patterns in positive affect – independent of ELA exposure – followed by multinomial logistic regression to examine associations between cumulative ELA exposure and trajectory membership. Mediation analyses tested whether positive affect trajectories explained links between ELA and psychopathology outcomes.

**Results:**

Four distinct positive affect trajectories emerged: High‐Stable, Declining, Persistently Low, and Volatile (*N* = 7,457). Higher ELA scores significantly predicted membership in all non‐high‐stable trajectories, with the strongest association existing for the Persistently Low group (β = .321, *p* < .001). Mediation analyses revealed that Persistently Low trajectory group membership significantly mediated the relationship between ELA and internalizing problems (indirect effect = 0.030, 95% CI [0.012, 0.056], *p* = .007), but not externalizing problems (*N* = 3,927).

**Conclusions:**

This study demonstrates that ELA shapes positive affect development through distinct, heterogeneous pathways rather than uniform effects, with persistently low positive affect representing a specific mechanism linking early adversity to later depression and anxiety. Findings suggest that targeting positive emotional experiences may be a promising intervention strategy for youth exposed to ELA.

## Introduction

Experiences of early life adversity (ELA) are unfortunately common and can profoundly shape youth mental health and development (Bhutta, Bhavnani, Betancourt, Tomlinson, & Patel, 2023; Gilgoff, Singh, Koita, Gentile, & Marques, 2020). Millions of children both nation‐wide and globally face ELAs such as physical abuse and neglect (Madigan et al., 2023; Sacks & Murphey, 2018); such adversities are linked to a host of developmental challenges including difficulties in social–emotional skills and increases in different forms of psychopathology (Doyle & Cicchetti, 2017). Particularly notable is the connection between ELAs and the emergence and development of internalizing symptoms in children and adolescents (Green et al., 2010). Meta‐analyses examining multiple forms of ELA find that youth with a history of ELA are not only more likely to develop depression and anxiety, but also have more severe presentations of these conditions, poorer treatment prognosis, and lower remission rates compared to youth without ELA (Espejo et al., 2007; Humphreys et al., 2020; LeMoult et al., 2020; Mandelli, Petrelli, & Serretti, 2015; Nanni, Uher, & Danese, 2012; Tyrka et al., 2013; Wiersma et al., 2009; Williams, Debattista, Duchemin, Schatzberg, & Nemeroff, 2016). Despite such findings, the pathways leading from ELA to depression remain unclear. Clarifying the developmental mechanisms linking ELA to depression is essential for designing targeted, developmentally informed interventions that improve outcomes for youth exposed to adversity.

The majority of past investigations on ELA and factors contributing to poor mental health have focused on threat and negative affect, finding that altered threat‐ and emotion processing of negative affect following ELA is associated with subsequent psychopathology (Glaser, Van Os, Portegijs, & Myin‐Germeys, 2006; McLaughlin, Hatzenbuehler, & Hilt, 2009; Pollak & Kistler, 2002). However, scarce work has explored potential alterations in *positive* affect (or lack thereof) after ELA. Positive affect can be conceptualized as emotions or feelings reflecting enthusiasm, energy, and pleasurable engagement with the environment such as happiness, joy, excitement, and contentment (Alexander et al., 2021; Clark, Watson, & Leeka, 1989; Watson, Clark, & Tellegen, 1988). Importantly, positive and negative affect represent largely independent dimensions of emotional experience rather than opposite poles of a single continuum (Watson et al., 1988; Watson & Tellegen, 1985). Factor analytic studies underscore that affective systems can be dissociated, that is, individuals may experience high levels of both positive and negative affect simultaneously, or show deficits in one domain while the other remains intact (Cacioppo et al., 1999; Diener & Emmons, 1984). This independence suggests that the mechanisms linking ELA to altered positive affect may be distinct from those involving negative emotional processing. The limited focus on positive affect in the literature on ELA and depression and anxiety is, however, notable given that self‐reported low positive affect and lower reactivity to positive stimuli have been connected to depression and, to a lesser extent, anxiety (Bylsma, Morris, & Rottenberg, 2008; Sequeira, Forbes, Hanson, & Silk, 2022; Trøstheim et al., 2020). For example, individuals with low positive affect are two to three times more likely to develop later depression (Rackoff & Newman, 2020; Wood & Joseph, 2010). In addition, low positive affect has been found to relate to anxiety disorders, with consistent connections found for social anxiety disorders (Kashdan, 2007; Naragon‐Gainey et al., 2009). Thinking more broadly, positive affect may be protective against other risk factors for depression and anxiety, as higher levels of these emotions can enhance the link between multiple resilience factors for depression such as social support, optimism, coping skills, and capacity to recover from negative events (Folkman, 2008; Khazanov & Ruscio, 2016; Southwick, Vythilingam, & Charney, 2005). Understanding the ways in which ELA may undermine positive affect is therefore essential to clarifying the role of positive affect in the developmental pathways to depression and anxiety.

ELA could negatively impact the development and expression of positive affect in several ways. First, ELAs could disrupt child‐caregiver attachment, affecting how children interpret social interactions and relationships (Cyr, Euser, Bakermans‐Kranenburg, & Van Ijzendoorn, 2010). Insecure or disorganized attachment may influence a child's ability to experience, recognize, and benefit from positive emotions and social connections by diminishing opportunities to learn normative social–emotional patterns (Palacios‐Barrios, Patel, & Hanson, 2024). Second, exposure to abuse, neglect, and other ELAs has been linked to dysregulated neuroendocrine and cortisol responsivity (Bunea, Szentágotai‐Tătar, & Miu, 2017; Shirtcliff, Hanson, Ruttle, Smith, & Pollak, 2024); these alterations could influence behavioral responses to stress and cascade to emotion dysregulation. Finally, ELAs may influence the development of brain regions that are critical for learning, memory, cognition, and behavioral control (Gorka, Hanson, Radtke, & Hariri, 2014; Hanson et al., 2010, 2012; Nweze, Banaschewski, et al., 2023; Nweze, Ezenwa, Ajaelu, Hanson, & Okoye, 2023). Taken together, these alterations to attachment, stress responsivity, cognition, and neurobiology could compromise abilities to experience, express, and draw meaning from positive emotions.

Consistent with these mechanisms, multiple studies have found that childhood maltreatment – one form of ELA – is associated with lower positive affect and emotions in adults (DePierro, D'Andrea, Frewen, & Todman, 2018; Etter, Gauthier, McDade‐Montez, Cloitre, & Carlson, 2013; Kuzminskaite et al., 2024; Myroniuk, Reitsema, de Jonge, & Jeronimus, 2024; Turiano, Silva, McDonald, & Hill, 2017; Xiang, Yuan, & Zhao, 2021). In child and adolescent samples, research in this area focused on positive affect has been limited. In young children (<4 years of age), laboratory tasks with caregivers designed to evoke positive affect or positive behaviors (e.g., cooperation and constructive play) have found lower levels of positive affect or behavior in children with high levels of ELA (Dadds, Mullins, McAllister, & Atkinson, 2003; Egeland & Sroufe, 1981; Egeland, Sroufe, & Erickson, 1983; Robinson et al., 2009). Additional support for the hypothesis that ELAs may impact positive affect comes from meta‐analyses finding that ELA leads to lower self‐report on psychosocial constructs related to positive affect such as hope, sense of purpose, and self‐concept (Hill, Turiano, & Burrow, 2018; Melamed, Botting, Lofthouse, Pass, & Meiser‐Stedman, 2024; Yarcheski & Mahon, 2016; Zhang, Wang, Liu, Feng, & Wei, 2023). Further, children who suffer ELAs often struggle with understanding and recognizing positive emotions (Bick, Luyster, Fox, Zeanah, & Nelson, 2017; Caouette, Cossette, & Hébert, 2023; Diaconu et al., 2023; Fries & Pollak, 2004; Koizumi & Takagishi, 2014; Pears & Fisher, 2005), or forecasting situations that might lead to positive emotional outcomes (Perlman, Kalish, & Pollak, 2008). Finally, ELAs also appear to impact behavioral responses to positive feedback and reward cues (Oltean, Șoflău, Miu, & Szentágotai‐Tătar, 2023) in adolescents (Hanson et al., 2017; Yazgan et al., 2021) and adults (Dillon et al., 2009). These findings collectively support the view that ELA undermines the capacity to experience, recognize, and respond to positive emotions.

While these past studies have provided important foundational insights, several key limitations exist that the current study seeks to overcome. First, emotional development across childhood and adolescence is dynamic, with youth learning how to better express, understand, and regulate their feelings throughout this time (Bailen, Green, & Thompson, 2019). Research has found decreases in positive emotions during the transition from childhood to adolescence (Larson, Moneta, Richards, & Wilson, 2002; Reitsema, Jeronimus, van Dijk, & de Jonge, 2022), necessitating a longitudinal understanding of these emotions' trajectories. Second, scarce research on child and adolescent samples has measured positive affect *directly*, instead using observational tasks or constructs related to positive affect (e.g., positive feedback processing; reward learning). Measuring positive affect via self‐report could be advantageous given that this emotion is a subjective, internal experience. Further, past work in *adult* samples that *have* directly measured positive affect has typically been cross‐sectional and relies on retrospective reports of ELA, therefore introducing recall bias and prohibiting an understanding of how differences in positive affect may change over time in relation to ELA. Past research has also not adequately considered behavioral heterogeneity in how positive affect may be altered following ELA exposure (Gee, 2021; Hanson, Kahhalé, & Sen, 2024). Group‐level analyses that examine mean differences may mask important subtypes of positive affect responsivity after adversity. Without person‐centered approaches that can identify distinct patterns of positive affect functioning (Linden & Hönekopp, 2021), our ability to understand individual risk and resilience factors remains limited, preventing us from distinguishing youth with early positive affect declines from those with delayed onset deficits, or from those who show late‐stage growth versus chronically low positive affect. Finally, previous projects have not typically connected differences in positive affect to symptoms of depression, anxiety, or other forms of psychopathology.

Motivated by these limitations, we leveraged the Adolescent Brain Cognitive Development (ABCD) Study® to investigate the longitudinal impact of ELA on positive affect and psychopathology during childhood and adolescence. We hypothesized that youth who had experienced greater levels of ELA would show altered positive affect trajectories compared to youth with lower adversity exposure. We believed that, upon examining these person‐centered positive affect trajectories, youth exposed to ELA would show early and consistent declines in positive affect, while others might exhibit declines later in development. Finally, we planned to examine relations between ELA, positive affect trajectories, and symptoms of psychopathology. We were interested in examining both *Internalizing* (e.g., Depression; Anxiety) and *Externalizing* (e.g., Aggressive Behavior, Conduct Problems) symptoms. This would allow us to understand if positive affect was a distinct mechanism for internalizing issues, rather than a general psychopathology risk factor. We predicted that differences in positive affect trajectories would account for the relation between cumulative ELA exposure and *internalizing* psychopathology symptoms, underscoring the potential role of positive affect in linking early adversity to later mental health outcomes.

## Method

### Participants and procedures

This work used data from the ABCD® study, an ongoing longitudinal project that aims to understand how various factors influence youth development. Data were derived from the ABCD 5.1 release and included a baseline visit and multiple follow‐up visits across the 4 subsequent years (see (Volkow et al., 2018) and the ABCD study website, https://abcdstudy.org/ for further details).

The ABCD study sampled individuals across 21 sites in the United States to reflect the nation's diverse socio‐demographic population. Researchers first created a catchment area which included public, private, and charter schools within 50 miles of a research site. Schools were then coded based on a series of factors (e.g., ethnic composition). The study used stratified sampling to randomly select schools from the catchment areas by presenting caregivers with recruitment materials. A total of 11,878 children were assessed at baseline (Karcher & Barch, 2021). Baseline data were collected in 2018 from a cohort of 9‐ to 10‐year‐old children and their caregivers.

### Ethical information

Each ABCD recruitment site obtained full assent and consent from the children and their parent(s)/legal guardian(s), respectively in accordance with local Institutional Review Boards. Our work was reviewed by the University of Pittsburgh's Institutional Review Board (ID: STUDY21020159). It was determined that our activity was not research involving human subjects as defined by U.S. Department of Health and Human Services and Food and Drug Administration regulations on 02/25/2021.

### Early life adversity (ELA)

ELA measures were identified from ABCD documentation and literature, reflecting categories from the commonly used Adverse Childhood Experiences (ACEs) scale (Felitti et al., 1998). A standardized cumulative risk score was derived from youth and caregiver reports across multiple baseline scales, as cumulative approaches have greater predictive power and overcome limitations of dimension‐specific methods, including ill‐defined boundaries, co‐occurring adversities, and lack of specificity in effects (for review and discussion, see Smith & Pollak, 2021). Assessing ELA during childhood also avoids retrospective recall biases inherent to adult self‐report (Colman et al., 2016). Items were drawn from eight ABCD scales including the Kiddie Schedule for Affective Disorders and Schizophrenia (K‐SADS), Family History Assessment, Neighborhood Safety, Parent Demographics, Family Environment Scale, Parental Monitoring, Children's Report of Parent Behavior, and Parent Adult Self Report. The methodology involved averaging items within each scale, *z*‐scoring responses at the scale level, standardizing scale averages across participants, and then averaging all *z*‐scores to generate a final cumulative risk score for each participant. Inclusion required completion of at least 75% of ELA scales. The mean for this standardized composite was −0.09 (standard deviation = 0.90) with a range from −1.09 to 12.61 (also see Figure S1). Additional information about this measure is detailed in our Appendix S1 and Table S1.

### Positive affect

Positive affect describes pleasant emotions that arise when we feel engaged and satisfied with our environment, including feelings like happiness, joy, excitement, enthusiasm, and contentment (Alexander et al., 2021). The Positive Affect Scale from the NIH Toolbox Battery (Salsman et al., 2013) was administered at different time points, but not at the baseline visit (i.e., after 6, 12, 18, 30, 36, and 42 months from the baseline visit), to evaluate positive emotions and affective well‐being in the past week. Respondents were provided with 9 positive affect items (e.g., ‘I felt calm’, ‘I felt delighted’) and indicated on a 3‐point Likert scale (1 = not true; 3 = very true) how much they felt that way in the last week. The scale showed high internal validity in past publications (Cronbach's alpha = .96). This measure was initially collected on *N* = 11,734 participants; the current study examined participants with six waves of data (from follow‐ups at 6 months, 1 year, 18 months, 30 months, 3 years, and 42 months).

### Psychopathology

Symptoms of psychopathology were measured via the Child Behavior Checklist (CBCL). The CBCL is a widely used parent‐report measure that assesses youth behavioral problems and psychopathology occurring in the past 6 months on a 3‐point scale (Achenbach, 2011). In the ABCD study, caregivers (primarily mothers) completed the CBCL during in‐person visits. The CBCL generates multiple scales that empirically capture different behavioral and developmental problems. The current study focused on the *Total Internalizing Score* (e.g., Anxious/Depressed, Withdrawn/Depressed) and *Total Externalizing Score* (e.g., Aggressive Behavior, Conduct Problems) scores. Both internalizing and externalizing scores represent composite measures created by summing items across their respective subscales, with higher scores indicating more severe psychopathology and behavioral problems. We used raw scores from the baseline and the Year 4 follow‐up visits. Focusing on both internalizing and externalizing outcomes would allow us to speak to whether lower positive affect was a specific risk for one form of psychopathology or rather a general psychopathology risk factor.

### Covariates

The following variables assessed at baseline were included as covariates in sensitivity models: participant sex assigned at birth (0 = male, 1 = female), race/ethnicity (1 = White, 2 = Black, 3 = Hispanic, 4 = Asian, 5 = Other), age in months, and recruitment site (dummy‐coded). Additional sensitivity models controlled for socioeconomic status, as measured by household income. In line with past work (Hair, Hanson, Wolfe, & Pollak, 2015; Hanson et al., 2013; Hanson, Chandra, Wolfe, & Pollak, 2011), the income variable was transformed by taking the midpoint of each income category and log‐transforming this value to normalize the highly skewed distribution and ensure robust statistical modeling.

### Statistical modeling

Data analysis consisted of three components: (1) person‐centered trajectory modeling of positive affect using time‐series analyses; (2) multinomial regression models; and (3) mediation modeling combining multinomial and ordinary least squares regressions.

#### Person‐centered trajectory modeling of positive affect

We cleaned and processed the longitudinal positive affect data by recoding invalid responses (e.g., 777, 999) as missing values and filtering out participants with four or more missing positive affect items. Only participants with complete data across all six waves were retained for analysis. Trajectory modeling using the R‐package *traj* (Sylvestre & Vatnik, 2014; Sylvestre, Vatnik, & Vatnik, 2025) involved calculating 19 time‐series indices assessing different aspects of longitudinal change patterns in individuals (Leffondré et al., 2004) including elementary measures of change (e.g., maximum, range, mean, standard deviation), linear modeling parameters (intercept, slope, *R* ^2^), measures of nonlinearity and inconsistency (e.g., curve length, rate of intersection with mean), measures sensitive to nonmonotonicity and short‐term fluctuations (first and second derivative statistics), and measures contrasting early versus later change. These measures are described in Appendix S2 and noted in Table S2. Outliers were identified and capped (not removed) using a probability‐based approach is a method conceptually similar to winsorizing (Nishiyama, 2018). Extreme values were defined as a threshold corresponding to a 0.3% probability and replaced with less extreme values allowing us to retain all observations while limiting the influence of extreme outliers on trajectory estimation. Next, highly correlated time‐series indices (>0.95) were removed to reduce redundancy. The remaining indices underwent principal component analysis to identify a subset that captured the most important trajectory features, which were subsequently used for k‐medoids clustering with 5,000 bootstrap samples and a maximum of 1,000 iterations. This analysis identified four distinct positive affect trajectory patterns. To determine the optimal number of clusters, we employed the Calinski–Harabasz criterion, which evaluates cluster quality by comparing the ratio of between‐cluster to within‐cluster variance (Caliński & Harabasz, 1974). Higher values indicate separation between trajectory groups while maintaining within‐cluster homogeneity. This person‐centered modeling was completed only for positive affect and was done independently of ELA and any other variables.

#### Multinomial regression

Models examined associations between cumulative ELA exposure and positive affect trajectory membership, controlling for relevant covariates (e.g., age, sex, race/ethnicity, socioeconomic status). These models estimated the likelihood of belonging to each trajectory cluster based on adversity exposure levels, with one cluster serving as the reference group (High‐Stability Positive Affect). Multinomial logistic regression was selected because the trajectory clusters represent a categorical outcome variable with more than two unordered categories.

#### Mediation

To test whether positive affect trajectory clusters mediated the association between cumulative ELA exposure and psychopathology outcomes, we employed an approach that combined multinomial logistic regression (for the categorical mediator of trajectory cluster membership) with ordinary least squares regression (for continuous psychopathology outcomes). We decomposed the total effect of ELA on psychopathology into direct effects (ELA → psychopathology) and indirect effects (ELA → trajectory clusters → psychopathology), accounting for the categorical nature of the mediator variable. Mediation models using alternative formulations of internalizing subscales are detailed in Appendix S3, Figure S2, and Table S3.

#### Data loss

While ABCD collected data on over 11,000 participants, we had smaller analytic samples for our multinomial regression and mediation models. One major source of data loss was missing positive affect measures from the six available assessments, as *N* = 4,277 participants did not have complete data on this questionnaire over time. This led to a final analytic *N* = 7,457 for our multinomial regression models examining associations between cumulative ELA exposure and positive affect trajectory cluster membership. With the mediation analyses, the analytic *N* = 3,927, with *N* = 3,530 participants being excluded for missing data from the CBCL at the 4‐year follow‐up of the ABCD study. Our supplement contains flow charts noting where participants were lost and how many participants were lost for different analyses (Figure S3), as well as missing completely at random (MCAR) analyses (also see Appendices S4 and S5).

#### Artificial intelligence generated content

Claude Sonnett 4.5 was used in October of 2025 to update the R syntax that was used to generate tables and graphs, specifically those in Appendix S3. The first author takes responsibility for the integrity of the content generated.

## Results

### Sociodemographic characteristics of the final sample are presented in Table 1

**Table 1:** Sociodemographic characteristics of study sample

Characteristic	Overall sample^a^
Age at Baseline (Months)	119.26 (7.47)
Sex	
Male	3,638 (52.2%)
Female	3,326 (47.8%)
Race/Ethnicity	
White	4,200 (60.3%)
Hispanic	1,279 (18.4%)
Black	655 (9.4%)
Other/Mixed	688 (9.9%)
Asian	142 (2.0%)
Household income	
<$25 K	304 (4.4%)
$25 K‐$49 K	736 (10.6%)
$50 K‐$99 K	2,617 (37.6%)
$100 K+	3,307 (47.5%)

^a^ Mean (*SD*); *n* (%).

#### Person‐centered trajectory modeling of positive affect

**Figure 1:**
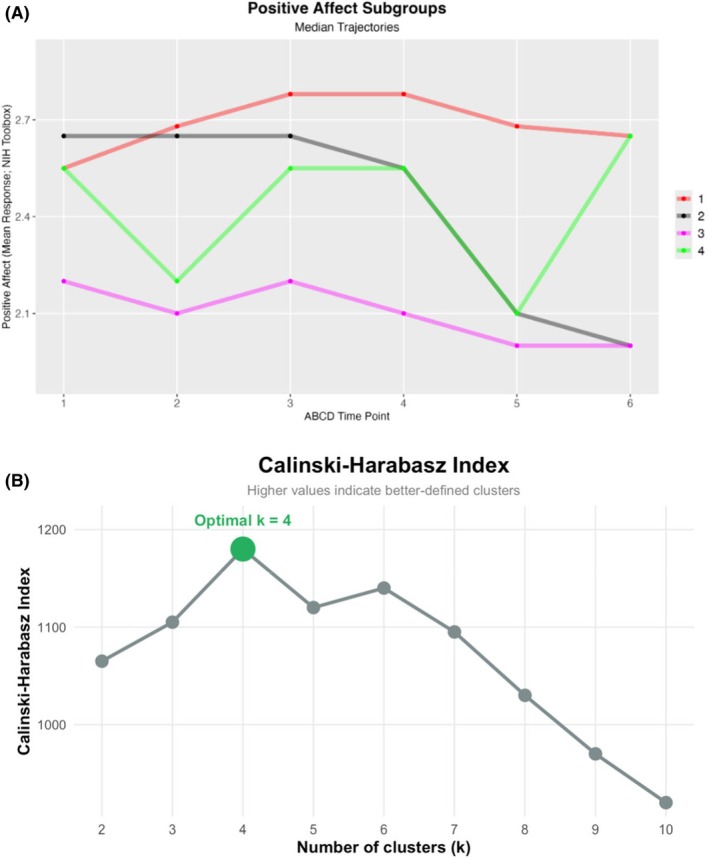
(A) Median positive affect trajectories across six ABCD study time points for four identified subgroups. Trajectories show positive affect mean response scores from the NIH Toolbox across developmental time points. Group 1 (red) shows high levels and primarily stability throughout development; Group 2 (black) demonstrates high levels initially, but then sharp declines in adolescence; Group 3 (magenta) exhibits persistently low levels; and Group 4 (green) displays a volatile pattern with significant fluctuations over time. (B) Calinski‐Harabasz Index values across different clustering solutions (*k* = 2–10). The optimal solution of four clusters was identified at *k* = 4 (peak value = 1184.84), indicating the most distinct and well‐separated trajectory subgroups

Using our non‐parametric trajectory‐based clustering approach, participants with complete follow‐up data (*N* = 7,457) were classified into four distinct clusters based on their trajectory of positive affect from ABCD time points 1 to 6 (Figure 1A). Calinski–Harabasz values peaked at *k* = 4 clusters (max value = 1188.84; Figure 1B), suggesting robust clustering structure. These groups were as follows: (1) High and Stable (*N* = 2,055; 27.6%), which represented the largest subgroup and showed high positive affect, without significant declines over development; (2) Declining (*N* = 1,846; 24.8%), which had initially high levels of positive affect, but then showed significant decline during early adolescence; (3) Persistently Low (*N* = 1,817; 24.4%), which was characterized by consistently lower positive affect levels through childhood and adolescence; and (4) Volatile (*N* = 1,739; 23.3%), where positive affect was more variable and unstable across development. Demographics for each subgroup are noted in Table 2.

**Table 2:** Sociodemographic characteristics by trajectory cluster

Characteristic	Trajectory cluster				*p*‐value^b^
	High‐stable	Declining	Persistently low	Volatile	
	*N* = 1,922^a^	*N* = 1,734^a^	*N* = 1,677^a^	*N* = 1,631^a^	
Age at baseline (months)	119.15 (7.43)	119.43 (7.48)	119.62 (7.45)	118.83 (7.50)	.013
Sex					
Male	1,066 (55.5%)	713 (41.1%)	955 (56.9%)	904 (55.4%)	<.001
Female	856 (44.5%)	1,021 (58.9%)	722 (43.1%)	727 (44.6%)	
Race/ethnicity					
White	1,212 (63.1%)	997 (57.5%)	994 (59.3%)	997 (61.1%)	.009
Hispanic	320 (16.6%)	347 (20.0%)	325 (19.4%)	287 (17.6%)	
Black	165 (8.6%)	187 (10.8%)	138 (8.2%)	165 (10.1%)	
Other/mixed	186 (9.7%)	174 (10.0%)	175 (10.4%)	153 (9.4%)	
Asian	39 (2.0%)	29 (1.7%)	45 (2.7%)	29 (1.8%)	
Household income					
<$25 K	68 (3.5%)	87 (5.0%)	70 (4.2%)	79 (4.8%)	<.001
$25 K‐$49 K	177 (9.2%)	196 (11.3%)	194 (11.6%)	169 (10.4%)	
$50 K‐$99 K	677 (35.2%)	685 (39.5%)	666 (39.7%)	589 (36.1%)	
$100 K+	1,000 (52.0%)	766 (44.2%)	747 (44.5%)	794 (48.7%)	

^a^ Mean (*SD*) or *n* (%).

^b^ Derived via One‐way analysis of means or Pearson's chi‐squared test.

#### Multinomial regression

**Figure 2:**
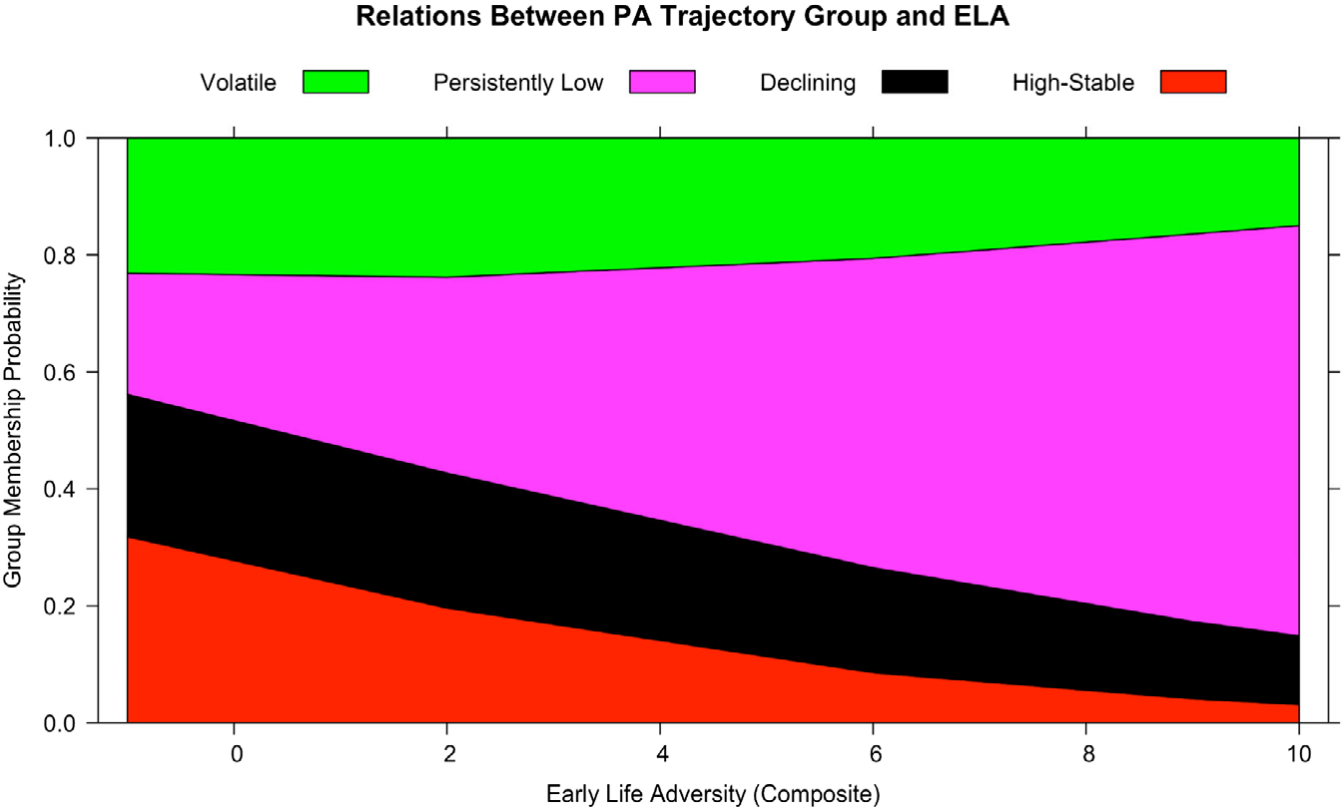
Relationship between positive affect trajectory group membership probability and early life adversity (ELA) composite scores. The stacked area chart displays the predicted probability of membership in four trajectory groups: Volatile (green), Persistently Low (magenta), Declining (black), and High‐Stable (red). As early life adversity increases, the probability of High‐Stable positive affect decreases while the probability of Persistently Low and Volatile patterns increases

Models revealed significant associations between the ELA composite score and positive affect trajectory membership. With the High and Stable group serving as the reference category, higher ELA scores were significantly associated with membership in all other trajectory groups. The strongest association was observed for the Persistently Low trajectory group (β = .321, *z* = 7.99, *p* < .001), followed by the Volatile trajectory group (β = .171, *z* = 4.00, *p* < .001) and the Declining trajectory group (β = .144, *z* = 3.34, *p* < .001) in models controlling for race/ethnicity, initial interview age (in months), sex assigned at birth, and recruitment site. Figure 2 displays the predicted probabilities of trajectory group membership across the range of ELA scores, illustrating that as ELA exposure increases, the probability of belonging to the High and Stable group decreases while probabilities of membership in the Persistently Low, Volatile, and Declining groups increase. Among youth with minimal adversity exposure, group membership was relatively evenly distributed. However, as adversity increased, the Declining trajectory emerged as the dominant pathway, increasing from 20% probability at low adversity to approximately 65%–70% at the highest adversity levels. Conversely, probability of High and Stable membership dropped to 4%–8.5% at high adversity. Results were similar for sensitivity models controlling for socioeconomic status, with ELA still relating to membership in the Declining trajectory group (β = .101, *z* = 2.22, *p* = .026), Persistently Low trajectory (β = .300, *z* = 7.04, *p* < .001), and Volatile trajectory (β = .146, *z* = 3.23, *p* = .001).

#### Mediation

**Figure 3:**
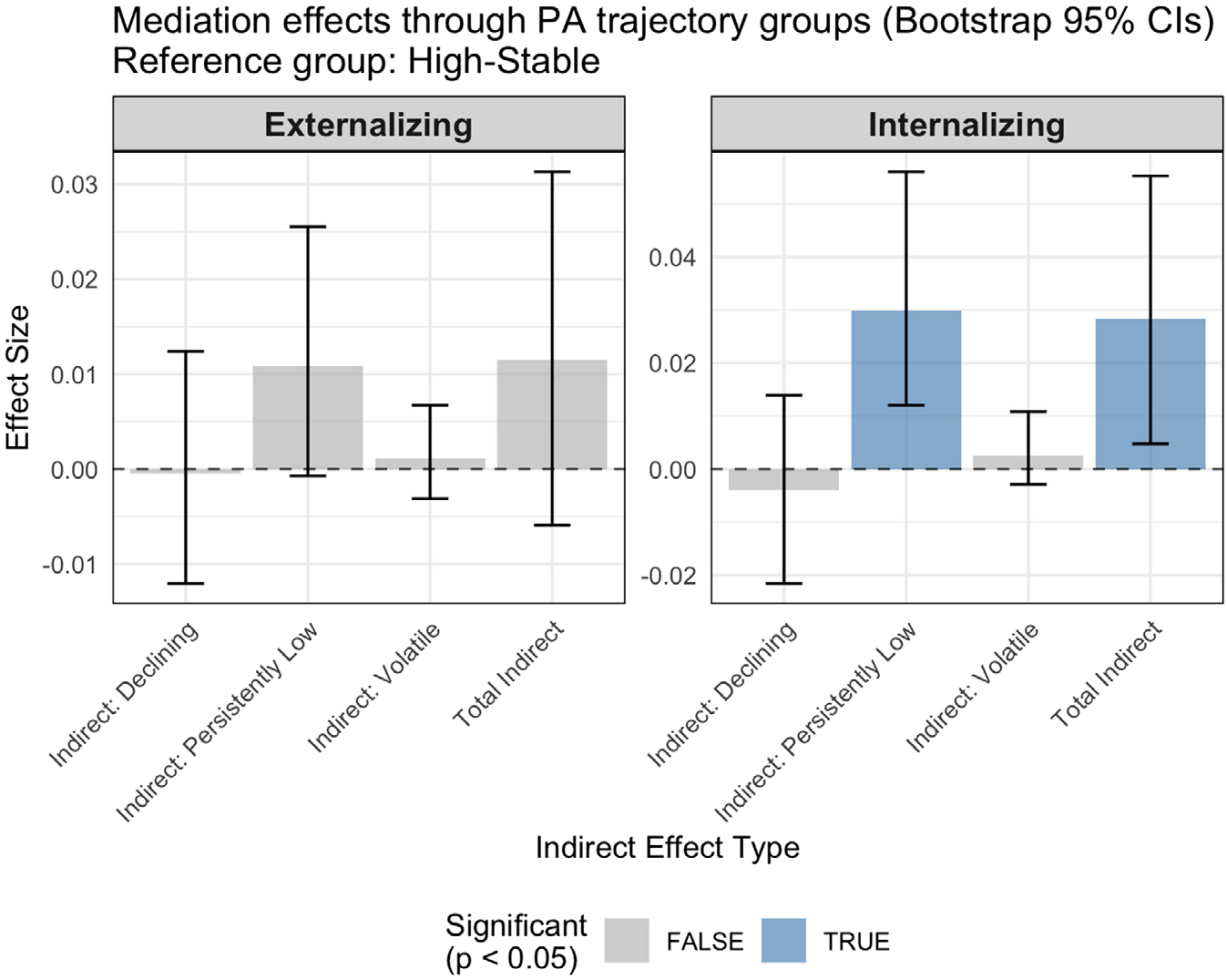
Mediation effects of positive affect trajectory groups on externalizing and internalizing outcomes with bootstrap 95% confidence intervals. The High‐Stable trajectory group serves as the reference category. Standardized effect sizes are shown for the indirect effect (ELA → Group X Group → Psychopathology symptoms) for each group (declining, persistently low, volatile). The total indirect effects are shown on the right portion of each panel. Blue bars indicate statistically significant effects (*p* < .05), while gray bars represent non‐significant effects. Significant mediation effects are observed for internalizing outcomes through persistently low positive affect trajectory pathways

We lastly examined if positive affect trajectory membership mediated links between ELA and internalizing or externalizing psychopathology. While ELA was directly related to externalizing issues (β = .338, *z* = 3.11, *p* = . 002), none of the individual trajectory pathways showed significant mediation effects for externalizing problems. The indirect effects through the Declining, Persistently Low, and Volatile trajectory groups were all non‐significant (all *p*'s > .101). For internalizing problems, ELA was directly related to internalizing issues (β = .248, *z* = 2.23, *p* = .026) and significant (partial) mediation was observed through the Persistently Low trajectory group (indirect effect = 0.030, 95% CI [0.012, 0.056], *z* = 2.679, *p* = .007). However, the indirect effects through the Declining and Volatile trajectory groups were not significant (*p*'s > .485). Figure 3 presents the indirect effects of ELA on externalizing and internalizing problems through each positive affect trajectory group.^1^ Table 3 lists the standardized coefficients with standard errors for relations between ELA and trajectory group, trajectory group and psychopathology, and ELA and psychopathology.^2^ Similar effects were found in sensitivity models controlling for socioeconomic status. No cluster membership mediated relations between ELA and externalizing psychopathology symptoms (all *p*'s > .106). For internalizing, when controlling for socioeconomic status, significant mediation was observed through the Persistently Low trajectory group (indirect effect = 0.033, 95% CI [0.013, 0.058], *z* = 2.747, *p* = .006).

**Table 3:** Mediation analysis: path coefficients.^a^ Standardized coefficients with standard errors^a^

Path	Internalizing problems^b^		Externalizing problems^b^	
	β	*SE*	β	*SE*
**(a) Paths**				
ELA → Declining	−.007	0.008	−.001	0.008
ELA → Persistently Low	.062***	0.008	.062***	0.008
ELA → Volatile	.021	0.009	.019	0.009
**(b) Paths**				
Declining → Outcome	.079***	0.222	.061***	0.190
Persistently Low → Outcome	.070***	0.223	.030	0.189
Volatile → Outcome	.017	0.207	.010	0.180
**(c′) Path**				
ELA → Outcome (Direct)	.036*	0.111	.058**	0.109

^a^ Reference group: High‐Stable positive affect trajectory.

^b^ ***p < .001, **p < .01, *p < .05.

## Discussion

In a large, nationally representative sample, we found that ELA was related to different trajectories of positive affect across childhood and adolescence, which in turn predicted internalizing psychopathology. Adversity experienced in childhood was related to a child's likelihood of having a low, declining, or volatile trajectory of positive affect compared to the high and stable positive affect trajectory. In fact, as adversity increases, the probability of being in the high and stable positive affect group was very low (4%–8.5%). Notably, the persistently low positive affect trajectory group was strongly associated with ELA and statistically explained the link between ELA and later internalizing symptoms. The person‐centered approach used here allowed us to isolate unique developmental trajectories that may mark risk or resilience after ELA, moving away from variable‐centered models (i.e., examining mean group differences) that have dominated past explorations.

Results revealed that positive emotions, or a lack thereof, may be an important mechanism linking ELA to later internalizing symptomatology including anxiety, depression, and withdrawal. Previous work on affect‐related impacts of ELA has uncovered alterations in threat and negative affect processing (Gorka et al., 2014; Hanson et al., 2015; Hanson & Nacewicz, 2021; Kraynak, Marsland, Hanson, & Gianaros, 2019), but such a focus on negative emotions discounts the fact that reduced positive emotions in the context of stress may also increase risk for depression and anxiety (Khazanov & Ruscio, 2016; Sequeira et al., 2022). The current findings align with emerging theories suggesting that ELAs disrupt core emotional and regulatory systems during sensitive developmental windows, including attachment processes, neuroendocrine functioning, and the maturation of brain regions involved in learning, memory, and behavioral control (Kennedy et al., 2021; Palacios‐Barrios et al., 2024; Shirtcliff et al., 2024). Notably, we found that positive affect statistically mediated links between ELA and internalizing – but not externalizing – problems, consistent with previous research finding that those experiencing low positive affect after stress are much more likely to develop later depression (Rackoff & Newman, 2020). Such findings underscore the possibility that ELA‐related disruptions in the development of positive affect may *specifically* heighten vulnerability to internalizing problems such as depression and anxiety, rather than contributing *broadly* to multiple forms of psychopathology. This differential specificity has important implications for understanding heterogeneity in mental health outcomes following ELA.

The heterogeneity in empirically derived positive affect trajectories may reflect differences in the emotion regulatory and neural circuits supporting positive emotional experiences. Meta‐analyses of group‐level findings (i.e., statistics examining mean differences) have found that ELA is related to lower positive affect (Lavi, Katz, Ozer, & Gross, 2019), impaired reward processing (Oltean et al., 2023), and lower responsivity in neural circuits critical for reward processing (Birn, Roeber, & Pollak, 2017; Hanson et al., 2016; Hanson, Hariri, & Williamson, 2015; Sacu et al., 2024; van Harmelen et al., 2014); for review, see (Hanson, Williams, Bangasser, & Peña, 2021). However, group‐level statistical approaches may mask important subtypes of trajectories after adversity. Future work could delve into neurobiological heterogeneity to differentiate subtypes of brain reactivity and functional connectivity in those exposed to ELA and examine whether and how such subtypes predict depression and anxiety symptomatology.

This study benefited from numerous strengths and points to several promising directions for future research. The large, geographically diverse sample enhances the generalizability of findings across populations and contexts, while the longitudinal design – spanning a sensitive developmental period from childhood through adolescence – captures a window of rapid emotional and neurobiological change. Although many youth exposed to ELA showed persistently low or declining positive affect, others maintained high levels of positive affect despite having experienced significant adversity. One critical next step for research is identifying the factors that support these more adaptive emotional trajectories. Recent work has highlighted certain neurobiological features such as variations in brain structure and functional connectivity as potential protective factors that predict positive adaptation across life domains (Hanson et al., 2019; Suarez et al., 2025). Future research should explore whether these neurobiological and psychosocial characteristics function as resilience markers, helping to buffer the impact of ELA and support mental health across development.

While our work has several strengths, there are several limitations to highlight. First, our approach to modeling ELA involved several methodological decisions that warrant discussion. We employed a continuous *z*‐score composite that averaged items within measurement scales, *z*‐scored at the scale level, and then averaged across scales. This approach offers several advantages, including greater sensitivity to variation in adversity levels and a parsimonious single measure that captures cumulative risk burden. However, like all cumulative risk approaches, our measure assumes equal impact of each adverse event and does not account for factors such as timing, severity, or individual perception of stress (Smith & Pollak, 2021). Alternative approaches, such as binary domain scoring where each adversity domain (e.g., physical abuse, emotional neglect) is coded as present or absent regardless of the number of items measuring that domain, may reduce measurement bias when domains are assessed with differing numbers of items (Breslin et al., 2025). Such approaches also allow for creation of multiple versions tailored to specific research questions, such as measures focused on traditional ACEs domains versus broader ELA conceptualizations that include exposure to community violence, lack of resources, and caregiver separation. While our continuous composite approach was well‐suited to examining dose–response relationships between adversity and developmental trajectories, future research could benefit from comparing findings across different operationalizations of ELA to understand how measurement choices influence conclusions about risk and resilience. Moreover, future research could incorporate dimensional ELA models and center youth perspectives on their subjective experiences to better understand how different ELA facets affect mental health (Kahhalé, Barry, & Hanson, 2023; Miller et al., 2018, 2025). Second, while the study assessments span several years, extended follow‐up periods into adulthood would provide important information about the persistence of observed patterns and their longer‐term mental health consequences. Third, the positive affect measure asked participants to reflect on feelings over the past week, which captures a shorter timeframe than measures assessing affect ‘in general’. Additionally, adolescent affect is known to fluctuate considerably in response to various situational factors (Reitsema et al., 2022). While this could raise questions about stability versus transient states, past evidence supports the stability and meaningfulness of these measurements (Diener et al., 2018; Watson et al., 1988), and our ability to consistently classify trajectory groups suggests these patterns capture meaningful developmental differences. Nonetheless, future research could examine convergence with daily diary methods, experience sampling approaches, or trait‐level affect measures to further characterize sources of variance and better distinguish developmental shifts from situational influences. Fourth, while positive affect was the focus of the current study, future studies should examine how ELA affects the balance between positive and negative emotional systems. The volatile trajectory group, in particular, may reflect disruptions in emotion regulation that affect both positive and negative emotional responses. Finally, as noted in Appendices S4 and S5, data were not missing at random. However, we believe this missingness could actually be underestimating our effects of interest, as excluded participants may be drawn from various ‘high risk’ demographics (e.g., lower income; families of color who face structural adversity, higher ELA).

Our work provides novel evidence that ELA shapes positive affect development in heterogeneous ways, with important implications for understanding risk and resilience pathways to depression and anxiety. The finding that persistently low positive affect partially mediates the connection between ELA and internalizing symptoms suggests a viable target for interventions. Early identification of persistently low positive affect may enable treatments focused on enhancing positive emotional experiences and building resilience before depressive symptoms fully emerge (Taylor, Lyubomirsky, & Stein, 2017). This work and future studies deploying nuanced, theory‐driven modeling of heterogeneity across ELA, affect, and psychopathology could identify vulnerable youth and optimize timing and targets for intervention.

## Ethical Considerations

Each ABCD recruitment site obtained full assent and consent from the children and their parent(s)/legal guardian(s), respectively in accordance with local Institutional Review Boards. Our work was reviewed by the University of Pittsburgh's Institutional Review Board (ID: STUDY21020159). It was determined that our activity was not research involving human subjects as defined by U.S. Department of Health and Human Services and Food and Drug Administration regulations on 02/25/2021.

**Key points**

**What's known?**

- Early life adversity (ELA) is established as a risk factor for depression, but prior research has predominantly focused on negative emotions rather than examining how ELA shapes positive emotional development across childhood and adolescence.

**What's new?**

- Using person‐centered trajectory modeling in 7,457 youth, we identified four distinct positive affect patterns: High‐Stable (27.6%), Declining (24.8%), Persistently Low (24.4%), and Volatile (23.3%). ELA exposure significantly predicted membership in all non‐stable trajectory groups. Critically, persistently low positive affect specifically mediated the relationship between ELA and internalizing problems (depression/anxiety) but not externalizing problems, revealing a targeted pathway to specific forms of psychopathology.

**What's relevant?**

- Early identification of youth with persistently low positive affect trajectories could enable targeted interventions focused on enhancing positive emotional experiences before depressive symptoms fully emerge.

## Supporting information


**Appendix S1.** Measurement of early life adversity.
**Appendix S2.** Trajectory features.
**Appendix S3.** Sensitivity analysis: mediation using alternative formulations of internalizing subscales.
**Appendix S4.** Missing data analysis: trajectory analyses.
**Appendix S5.** Missing data analysis: mediation analyses.
**Table S1.** Descriptions for each ELA item.
**Table S2.** Trajectory features used in clustering analysis.
**Table S3.** Items removed: reduced overlap 21‐item scale.
**Figure S1.** Distribution of ELA *Z*‐score.
**Figure S2.** Mediation using alternative formulations of internalizing subscales.
**Figure S3.** Missing data diagram.

## Data Availability

Data used in this study were obtained from the Adolescent Brain Cognitive Development (ABCD) Study (https://abcdstudy.org), held in the NIMH Data Archive. The ABCD data used in this report came from the 5.1 release (information available here: https://doi.org/10.15154/cqdy‐5453).

## Endnotes

1 The High and Stable group again served as the reference category.

2 Analyses were completed in 3,927 participants, as 3,530 participants did not have data from the CBCL at the 4‐year follow‐up timepoint of the ABCD study.
